# Dementia in low-income and middle-income countries: Different realities mandate tailored solutions

**DOI:** 10.1371/journal.pmed.1002271

**Published:** 2017-03-28

**Authors:** Cleusa Pinheiro Ferri, K. S. Jacob

**Affiliations:** 1 Department of Psychobiology, Universidade Federal de São Paulo, Sao Paulo, Brazil; 2 Department of Psychiatry, Christian Medical College, Vellore, India

## Abstract

In a Perspective, Cleusa Ferri and K. S. Jacob discuss the assessment, recognition, and care of people living with dementia in low- and middle-income countries.

The ageing of populations is the most significant social transformation of the 21st century [1] and has highlighted the importance of age-related conditions such as dementia, which has been recognised across regions, countries, and cultures. The number of people living with dementia has been increasing and is estimated to reach 75 million worldwide by 2030, with the majority of these individuals living in low-income and middle-income countries (LMICs) [2]. The assessment, recognition, and care of people living with dementia in LMICs are complex issues. Dementia is often seen as part of the ageing process, and even when recognized, there still remain problems related to stigma, lack of resources for the adequate care of people with dementia (PWD), variations in the way the condition is assessed and perceived, and how it is addressed in noncommunicable disease (NCD) policies and prevention strategies.

## Prevalence

Ageing across the world’s populations is not a homogenous and uniform process. Over the next 15 years, the number of older people is projected to increase by 71% in Latin America and the Caribbean, 66% in Asia, 64% in Africa, 47% in Oceania, 41% in North America, and 23% in Europe [1]. The differences in the base populations and the rates of growth and longevity mean there will be wide variations between regions.

While the estimates of people living with dementia across regions and countries show a clear increase in numbers, differences in prevalence rates have been reported. A recent systematic review of the global prevalence of dementia [3] showed that age-standardized prevalence (to the population of western Europe) varied from 2.1% in sub-Saharan Africa to 8.5% in Latin America. While true differences in population prevalence exist (attributed to differing genetic and environmental factors, life expectancy, duration with disease, and age-specific incidence), variations in prevalence data may also be due to the use of different data collection procedures (one stage/two stage), assessment schedules, diagnostic criteria, and cultural conceptions of the condition.

Initiatives such as the 10/66 Dementia Research Group employed a standardised methodology to assess the prevalence of dementia in several LMICs (e.g., countries in Latin America, China, and India) [4]. However, an analysis of data from Vellore, India, which was part of the consortium, demonstrated wide variations in prevalence depending on the diagnostic criteria used. The prevalence of dementia among people aged 65 years and over was 63.4% according to the Geriatric Mental State Examination, 21.2% with the original 10/66 diagnostic algorithm, 10.6% using the education adjusted 10/66 algorithm, and 0.8% with the Diagnostic and Statistical Manual IV (DSM-IV) criteria [5]. While information variance (e.g., different informants and interview schedules) is a common reason for differences in prevalence, variations in diagnostic criteria contribute to significant differences in the threshold for the condition, number, and the type of patients identified and pose a major problem for cross-national comparisons. In addition, DSM-5 has made significant changes to its diagnostic criteria for dementia (major neurocognitive disorder). For example, it demands comparison of the individual’s performance in neuropsychological tests with population norms, adjusted for age, education, and cultural background. The absence of population norms and the lack of resources to conduct detailed neuropsychological tests in LMICs will complicate its use and prevent comparison.

## Prevention

Recent studies have reported a decline in the prevalence of dementia in high-income countries [6,7]. It has been suggested that this decline might be the result of changes in the profile of risk factors for dementia, suggesting that dementia may, at least partially, be preventable. Considering the costs of dementia care, primary prevention is likely to be the cheapest way to reduce the projected impact of dementia in future generations [8]. Evidence for the effectiveness of prevention programs that focus on local contexts and modifiable risk factors needs to be strengthened in order to design effective interventions and appropriate public health policies.

## Recognition

Dementia is under-recognised, underdisclosed, undertreated, and undermanaged, particularly in LMICs, with rates varying between countries [9]. Symptoms of dementia are considered a part of normal ageing in many LMICs and are not perceived as requiring medical care. The lack of awareness and stigma also results in a failure to seek help and treatment [9]. Many caregivers do not complain of problems, although their relatives may have significant cognitive impairment. The high tolerance to such symptoms and disability is often due to families’ low expectations of their older relatives [5], which results in lower recognition rates, as deterioration of social and occupational functioning is mandatory for a diagnosis of dementia by DSM criteria.

A strategy of employing community health workers to identify mental illnesses in general and dementia in particular in resource-poor settings has been recommended [9]; however, it has been found that this strategy leads to a very high false positive rate [10,11]. The reasons for this rate include the fact that disorders with low prevalence at the community level cannot be diagnosed accurately unless a referral system is in place. Such a tiered organization (e.g., health worker, public health nurse, and physician), which screens, confirms, and refers people at risk up a pathway of care, is required to improve the overall accuracy of the system [12]. The failure to put in place such a diagnostic system, which employs health personnel to filter cases at multiple levels, has contributed to the failure of community psychiatry programs across LMICs to identify and manage mental disorders, including dementia.

## Care

The progressive nature of the disease alters the care needs of people living with dementia over time and requires continual assessment and tailored approaches to changes in clinical problems and presentations [9]. While more responsive and flexible health care models that focus on task shifting and task sharing have been discussed in high-income countries (HICs), the challenges are even more complex with the limited resources in LMICs. The identification of the critical role of local and social determinants to disability [13] and the paucity of medical interventions to reverse pathology in dementia mandate the need for psychosocial support, accommodation, and nursing care as the main stay of management [9]. The lack of formal and institutional care for dementia in LMICs places a huge burden on relatives and carers.

Reduced public health expenditure and increased reliance on the private sector have limited access to universal health care in many LMICs and mean that families and individuals have had to bear the burden of dementia costs themselves [14]. Overstretched and under-resourced public health systems, low staff morale, poor training, and limited expertise in mental health result in poor care for older people, particularly those with dementia. There is a need to integrate social and health intervention programs, as well as formal and informal service arrangements to optimize care for older people.

## Conclusion

The relative success of programmes in HICs and their replication in successful projects in LMICs do not necessarily guarantee the possibility of scaling them up or their cost-effectiveness when rolled out to larger populations. The distinctive context of care for older people in LMICs argues for a need to tailor solutions to the prevalent reality. Each country will have to find its best response within the context of its own limitations and possibilities, but it should be based on knowledge of local resources and burden of disease so that its impact can be evaluated and the most effective and sustainable response be delivered.

## Abbreviations

DSM: Diagnostic and Statistical Manual
HIC: high-income country
LMIC: low-income and middle-income country
NCD: noncommunicable disease
PWD: people with dementia
